# A deep learning model for detection of cervical spinal cord compression in MRI scans

**DOI:** 10.1038/s41598-021-89848-3

**Published:** 2021-05-18

**Authors:** Zamir Merali, Justin Z. Wang, Jetan H. Badhiwala, Christopher D. Witiw, Jefferson R. Wilson, Michael G. Fehlings

**Affiliations:** 1 Division of Neurosurgery, Department of Surgery, University of Toronto, 149 College Street, Toronto, ON M5T 1P5 Canada; 2 Division of Neurosurgery, St. Michael’s Hospital, 30 Bond Street, Toronto, ON M5B 1W8 Canada; 3 Division of Neurosurgery, Krembil Neuroscience Centre, University Health Network, 399 Bathurst Street, Suite 4W-449, Toronto, ON M5T 2S8 Canada

**Keywords:** Neurology, Neurological disorders, Diagnostic markers

## Abstract

Magnetic Resonance Imaging (MRI) evidence of spinal cord compression plays a central role in the diagnosis of degenerative cervical myelopathy (DCM). There is growing recognition that deep learning models may assist in addressing the increasing volume of medical imaging data and provide initial interpretation of images gathered in a primary-care setting. We aimed to develop and validate a deep learning model for detection of cervical spinal cord compression in MRI scans. Patients undergoing surgery for DCM as a part of the AO Spine CSM-NA or CSM-I prospective cohort studies were included in our study. Patients were divided into a training/validation or holdout dataset. Images were labelled by two specialist physicians. We trained a deep convolutional neural network using images from the training/validation dataset and assessed model performance on the holdout dataset. The training/validation cohort included 201 patients with 6588 images and the holdout dataset included 88 patients with 2991 images. On the holdout dataset the deep learning model achieved an overall AUC of 0.94, sensitivity of 0.88, specificity of 0.89, and f1-score of 0.82. This model could improve the efficiency and objectivity of the interpretation of cervical spine MRI scans.

## Introduction

Degenerative cervical myelopathy (DCM) is a condition that results in progressive non-traumatic compression of the cervical spinal cord^1^. Globally, DCM is the most common cause of spinal cord impairment and can result in significant decline in function and quality of life among affected patients^2^. Magnetic Resonance Imaging (MRI) plays a central role in the diagnosis of DCM^3^. An MRI showing compression of the cervical spinal cord by extrinsic tissues such an intervertebral disk, ligament, or bone is highly suggestive of a clinical diagnosis of DCM and often results in referral to a spine surgeon^4^.

Cervical spine MRI scans are often acquired in a primary care or emergency room setting and interpreted manually by radiologists. There is growing recognition that computer models may assist in the initial interpretation of medical imaging studies and rapidly flag studies with pathologic findings^5–9^. Deep convolutional neural networks (deep learning methods) have shown promise in this area and have been tested in a variety of pathology categories such as pulmonary nodule detection with Computed Tomography (CT) and intracranial bleed detection with CT^10,11^.

Researchers have made use of convolutional neural networks (CNNs) for automated segmentation of spinal imaging^12,13^. In addition, previous studies have utilized computer vision methods, including CNNs, to extract quantitative parameters from cervical spinal cord MRI scans^14,15^. No studies to date, however, have attempted to use deep learning methods to detect spinal cord compression in a population of patients with DCM.

In the present study, our aim was to develop a novel deep learning model to detect cervical spinal cord compression in patients with DCM in T2 weighted MRI scans. As DCM is a heterogenous condition we aimed to develop a model that would have similar performance in patients with various demographics, disease characteristics and on images gathered with various MRI scanners. After developing a model we attempted to gain insights into how the model functioned using analytic techniques.

## Materials and methods

### Data acquisition

This study involved retrospective analysis of prospectively collected magnetic resonance imaging (MRI) studies from patients with DCM enrolled in the AO Spine CSM North America (CSM-NA; ClinicalTrials.gov NCT00285337) or AO Spine CSM International (CSM-I; Clinical Trials.gov NCT00565734) clinical studies^16,17^. The NCT00285337 and NCT00565734 clinical studies received institutional research ethics board approval at each respective site (Table S1). All patients were over the age of 18 and provided informed consent prior to enrolment in the NCT00285337 and NCT00565734 clinical studies, which included consent for post-hoc analyses of study data. This study was approved by the University Health Network Research Ethics Board and conducted in accordance with all relevant institutional and regulatory guidelines.

Patients were enrolled if they met eligibility criteria as follows: (1) Age 18 years or older; (2) imaging evidence of cervical spinal cord compression; (3) symptomatic DCM with one or more signs of myelopathy; and (4) no prior cervical spine surgery. Exclusion criteria were asymptomatic DCM, active infection, neoplastic disease, rheumatoid arthritis, trauma, ankylosing spondylitis, or concomitant lumbar stenosis. All patients had a pre-operative MRI scan then underwent surgical decompression of the cervical spine, with or without instrumented fusion. Patients were subsequently followed for 2 years after surgery.

All patients in this study had imaging evidence of cervical spinal cord compression. However, the spinal cord compression typically affected only a portion of the cervical spine. In each MRI scan there were some spinal levels that were compressed and other spinal levels that were not compressed. Thus, we were able to obtain images of spinal cord compression and non-compressed spinal cords from this patient cohort for model training.

**Figure 1:**
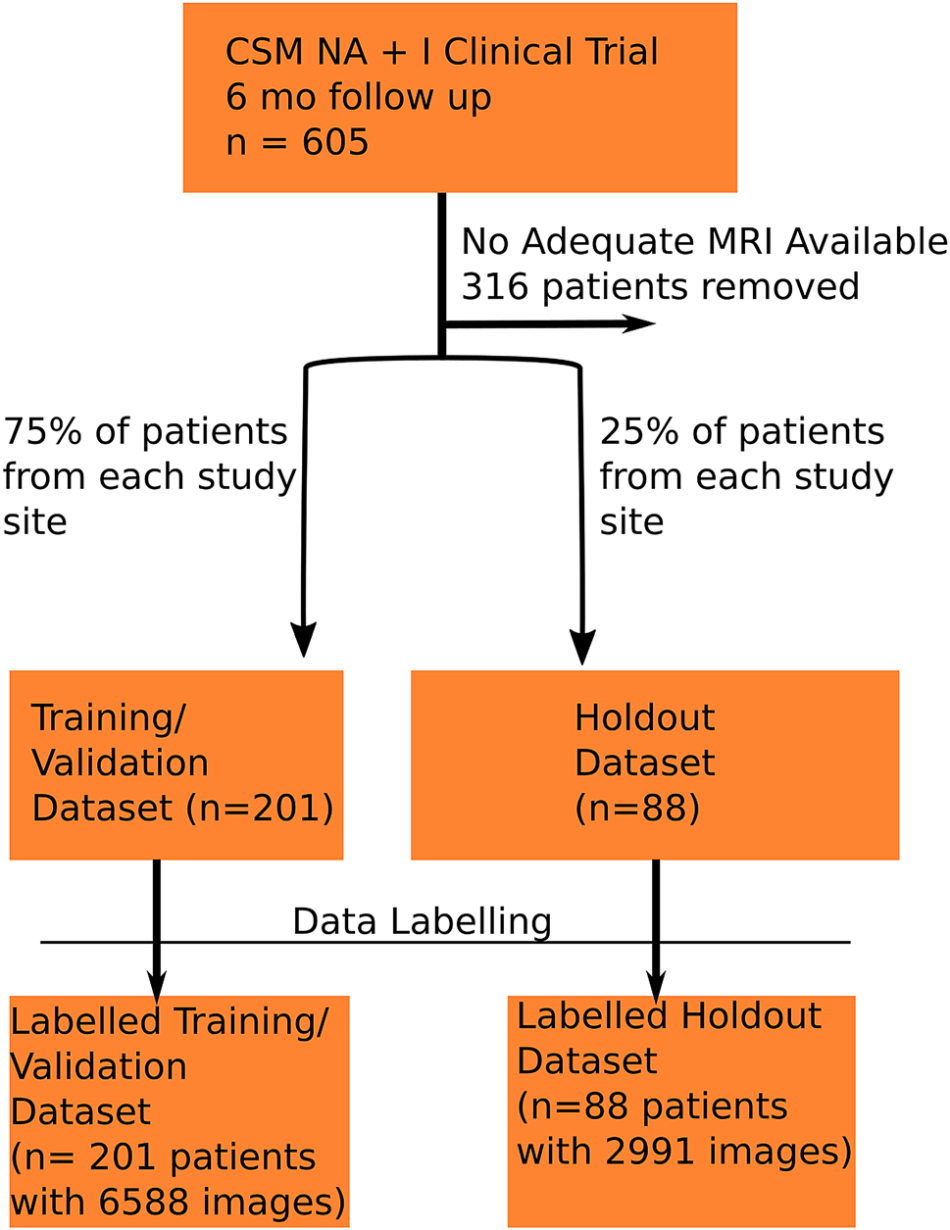
Consort diagram showing the process of data acquisition and partitioning into training/validation and holdout datasets. CSM-NA—Cervical Spondylotic Myelopathy North American Clinical Trial. CSM-I—Cervical Spondylotic Myelopathy International Trial.

Two patient cohorts were defined for model development and validation (Fig. 1):

1. *Training/Validation Dataset* Used for model development, training, and validation. Seventy-five percent of patients from each site were randomly chosen and allocated to the training/validation dataset.
2. *Holdout Dataset* Used for model testing and assessment of external validity. The remaining 25% of patients from each site were allocated to the holdout dataset.

For each patient the MRI study was collected in DICOM format. In addition, baseline clinical data was collected including demographic information (age, gender, weight, height), and the modified Japanese orthopedic association (mJOA) score^18^. The mJOA score is a standard measure of degenerative cervical myelopathy symptoms that measures symptom severity in the upper and lower extremities as well as sphincter function. The mJOA score is commonly used in surgical decision-making and is associated with post-operative outcomes^19^. The baseline clinical data, mJOA score, and MRI image parameters were compared between the training/validation and holdout dataset. We used t-tests to compare continuous variables and X^2^ tests to compare proportions. These statistical comparisons were completed using RStudio v1.3.

### Data pre-processing

A data pre-processing protocol was developed and was applied to the training/validation as well as holdout datasets. The axial T2-weighted non-fat saturated (aT2w) sequence from each MRI study was anonymized to remove patient identifiers using a freely available software package, DicomCleaner (TM)^20^. A linking library was created to reference the anonymized MRI studies to the original dataset.

The MRI scanner manufacturer and acquisition parameters, including voxel size and slice thickness for the T2-weighted axial sequence, were collected. The aT2w sequence for each patient was converted into a series of JPEG images. We down-sampled each image to a 299 × 299 image. We then normalized pixel values between 0 and 1 and stored each image in a 299 × 299 × 3 array, where each value in the array represented the pixel intensity in the red, green, and blue channels to construct the grayscale MRI image. All image pre-processing was completed using Python v3.6 with the pydicom v1.0 and scikit-image v0.18 packages. MRI scans were gathered at different sites and there was some variability in the number of slices between MRI scans. We did not normalize the number of slices so that each patient would have a consistent number of slices but rather allowed patients to have differing numbers of slices. This was done to preserve heterogeneity in the dataset.

### Data labeling

**Figure 2:**
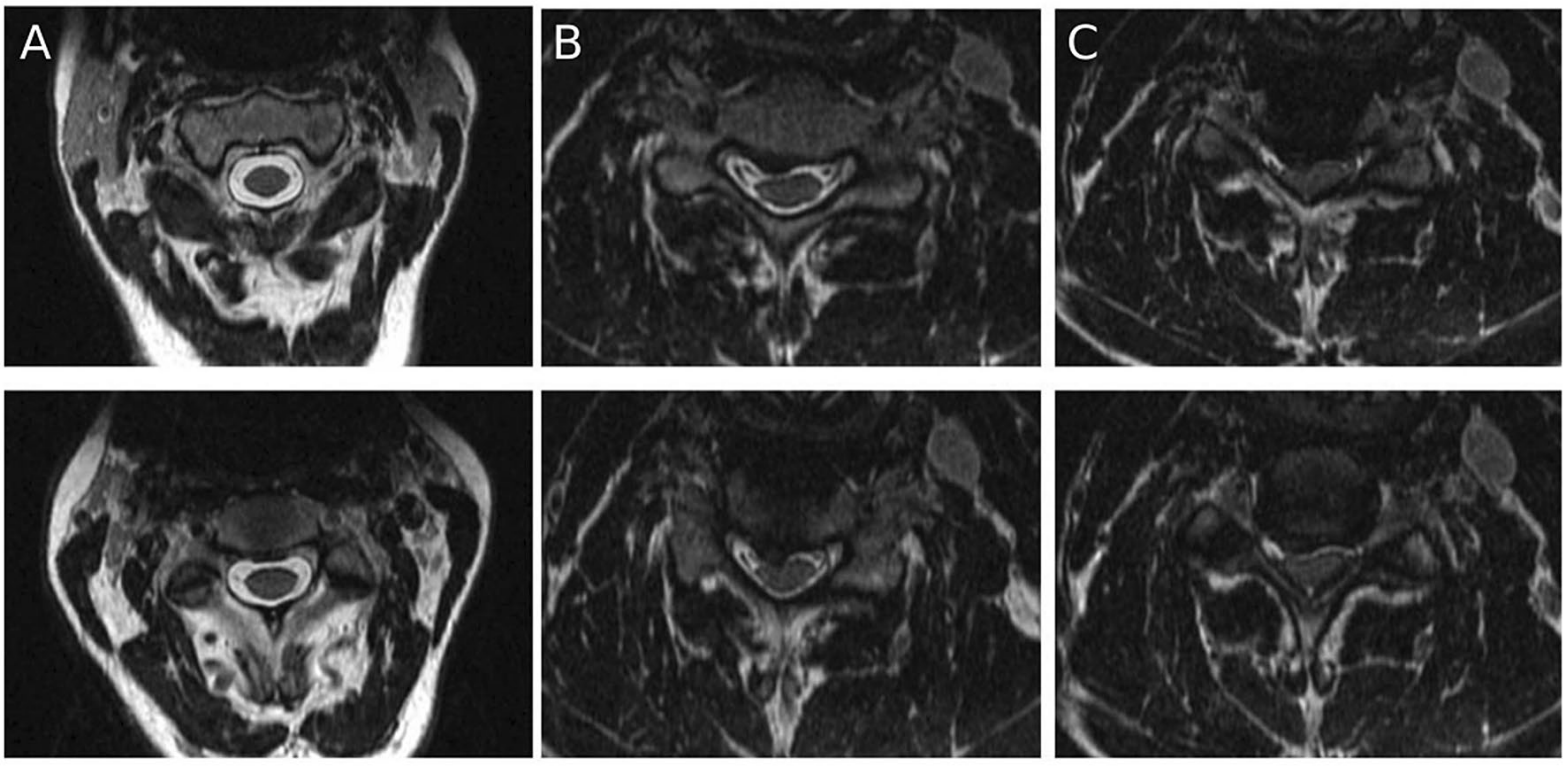
Representative axial T2-weighted MRI images showing (**A**) no spinal cord compression, (**B**) partial spinal cord compression, and (**C**) circumferential spinal cord compression.

Two senior neurosurgical residents, each with > 4 years experience interpreting cervical spine MRI scans, independently examined each axial T2-weighted image from the training/validation and holdout dataset. The labelers were given the full resolution (prior to down-sampling to 299 × 299) JPEG images to review with no time limit per image. Spinal cord compression was defined as any indentation, flattening, torsion, or circumferential compression of the spinal cord parenchyma from extrinsic tissues (disk, ligament, or bone) (Fig. 2)^3^. Raters were provided written directions and were instructed to label images in a binary fashion. Any image with evidence of partial or circumferential spinal cord compression were labelled ‘compressed’ and images in which these qualitative criteria were not present were labeled ‘non-compressed’.

The labels assigned by the two labelers were compared and the Cohen’s kappa metric of inter-rater reliability was generated. In cases where the two-labelers disagreed, both labelers reviewed these images together and a final label was decided upon after a period of discussion. The final set of labels after cases of disagreement were reconciled was taken as the ground-truth set of labels.

### Model training

The convolutional neural network architecture ResNet-50 was used in this study^21^. ResNet-50 is a residual CNN that is commonly used for image classification tasks. A residual CNN makes use of blocks of layers (residual blocks) that function together to create a very deep CNN that has been shown to perform well on image classification tasks^21^. The initial weights for the network were the transfer learning weights that were developed during the ImageNet competition^22^. Transfer learning is a process in which initial model weights are specified from a model that has been trained on another image classification task. The initialization weights from another image classification task can be easier to tune than randomly assigned weights because earlier layers in the CNN focus on detection and combinations of simple image features such as edges, which can generalize across different classification tasks. Transfer learning can potentially improve model training and performance on a smaller dataset^23^.

**Figure 3:**
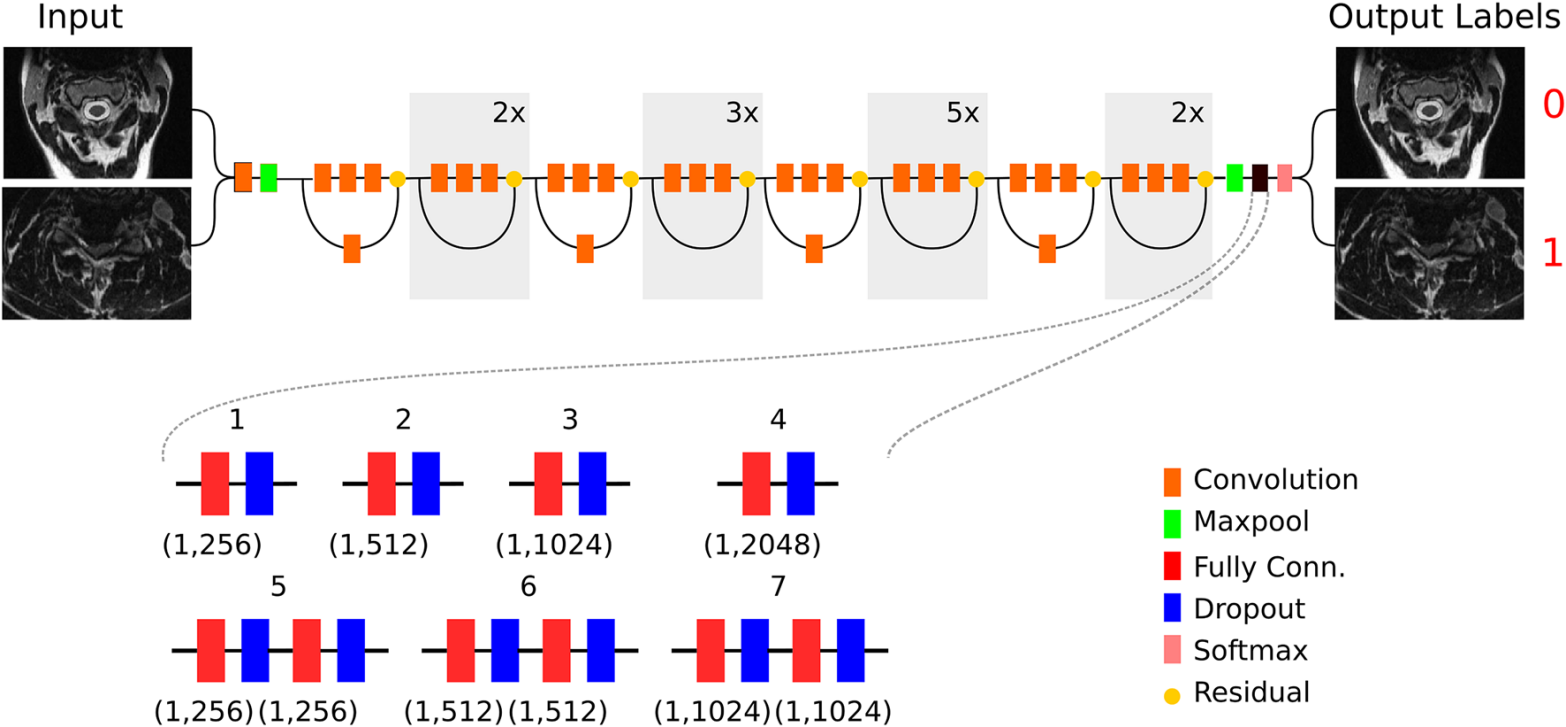
Overview of the convolutional neural network model architecture. The convolutional layers (orange) were derived from the Resnet-50 model, while the fully connected layers were modified for our classification task. Seven separate model configurations were tested as shown.

The fully connected layers from the ResNet-50 network were replaced by a set of fully connected layers with randomly initialized weights. A greater number of neurons in the fully connected layers can potentially increase the ability of the network to discriminate between features at the cost of an increased number of parameters and greater potential for overfitting^24^. In addition, multiple stacked fully connected layers can increase model performance in certain datasets^25^. Drop out layers can reduce the chance of model overfitting during training^26^. We tested a variety of network configurations to determine which would function best for our dataset. Models 1, 2, 3, and 4 had a single fully connected layer with 256, 512, 1024, and 2048 neurons respectively and a single dropout layer with 30% dropout. Models 5, 6, and 7 had two fully connected layers with 256, 512, and 1024 neurons each with two dropout layers of 30% each. The network architectures are described schematically in more detail in (Fig. 3). We used 30% dropout in each network architecture because we found this level of dropout to function well in initial testing.

The Training/Validation Dataset with associated labels was shuffled and split such that 75% of patients were used for model training and 25% of patients were used to validate model performance during training. During model training we used Adam as an optimizer with an initial learning rate of 0.0001, batch size of 16, momentum of 0.9, and no learning rate decay. We monitored binary cross-entropy loss in the validation set during training. To account for imbalance in compressed and not-compressed images, we used a weighting factor when calculating binary cross-entropy loss. The compressed class was given a weight of 3.8 and the not-compressed class a weight of 1. During model training a random search strategy was adopted for tuning hyper-parameters (learning rate, and momentum). Each network configuration was trained using the Training/Validation Dataset and training was continued until the weighted binary cross-entropy loss on the validation set had not decreased for 10 epochs. During initial trails we found that validation loss sometimes plateaued early but continued to decrease after 50 epochs. We thus allowed all models to train for at least 50 epochs before early stopping. All training was carried out using the Keras v1.3 package with TensorFlow v1.2 backend within Python 3.6. After training, each model configuration was compared using binary cross-entropy loss and accuracy on the validation set and the best performing model was chosen for further testing.

### Model testing and external validity

The best performing model architecture was carried forward for testing on the holdout dataset. The model was applied to each image in the holdout dataset and a predicted class was generated for each image. The predicted classes generated by the model were compared to the ground-truth labels generated by the human labelers across the entire training dataset and the following summary statistics were generated: area under the receiver operating characteristic curve (AUC), sensitivity, specificity, and f1 score. Performance metrics were calculated using Python 3.6 with the scikit-learn v0.2 package during a default threshold of 0.5.

The model’s performance was further assessed for each patient. For each patient’s MRI scan the set of predicted classes generated by the model was compared to the ground-truth labels generated by the human labelers. The AUC was calculated for each patient. The average AUC for all patients was calculated. Patients were then stratified by age (< 40, 40–65, or > 65), baseline mJOA (18, 15–17, 12–14, < 12), location, as well as MRI scanner type, and the average AUC within each of these subgroups was calculated. The AUC for each subgroup was compared to the AUC for all patients using the DeLong test with a significance level of p < 0.05.

Class Activation Maps.

Deep learning models have been characterized as a “black box” because it can be difficult to understand how a model makes a classification^27^. This can pose a problem when CNNs are applied to medical imaging tasks when a model learns to make a classification based on an irrelevant part of the image^28^. Model visualization with class activation maps (CAMs) can help identify the image features that are associated with activation of a particular class^29^. Class activation maps project the weights of the output layer back to the final convolutional layer (the last layer with spatial information) to determine which image regions, if changed, would most modify the probability of an image belonging to a particular class^29^. We generated CAMs for randomly selected images that were classified correctly (true positives) and classified incorrectly (false negatives). The CAMs for the true positive images were examined to see which image features corresponded to activation of the compressed class. In the false negative images, in which the CNN missed spinal cord compression, we examined the CAMs to gain insight into the features that corresponded to activation of the not-compressed class in misclassified images. The Keras-vis package in Python 3.6 was used to generate the CAMs^30^.

### Public code repository

Our code is made public at https://github.com/zamirmerali/dcm-mri

## Results

### Demographics

We identified a total of 289 patients with available MRI scans that could be used for model development. The Training/Validation Dataset was comprised of 201 patients with 6588 individual training images. The Holdout Dataset was comprised of 88 patients with 2991 individual images. Demographic information from each dataset is seen in (Table 1). Patient age, gender, baseline mJOA, MRI scanner manufacturer, and MRI image parameters, did not significantly differ between the Training/Validation Dataset and the Holdout Dataset.

**Table 1:** Demographics of patients and scanner parameters in the training/validation and holdout datasets.

Dataset	Training/Validation (n = 201)	Holdout (n = 88)	p-value
Age (median)	55	56	0.65
Gender (male)	63%	66%	0.53
Baseline mJOA (median)	13	13	0.72
MRI Scanner Manufacturer	GE Medical Systems (n = 98) Siemens (n = 12)	GE Medical Systems (n = 92) Siemens (n = 66) Philips Medical Systems (n = 21)	0.12
Slice thickness median (range)	3 (2–5) mm	3 (2–5) mm	0.82
Voxel size median (range)	0.3516 (0.2539–0.7813) mm	0.3516 (0.2539–0.7813) mm	0.65

### Inter-rater reliability

Two independent raters reviewed the 6588 images in the Training/Validation Dataset as well as the 2991 images in the Holdout Dataset and identified images with evidence of partial or circumferential spinal cord compression. Concordance between the two raters on the training/validation dataset had a Cohen’s κ = 0.82 and on the holdout dataset a Cohen’s κ = 0.83 (Table 2).

**Table 2:** Inter-rater reliability between labelers on the training dataset.

Dataset	Training/Validation		Holdout	
	Compressed	Not Compressed	Compressed	Not Compressed
Labeler 1	1542/6588 (23.4%)	5046/6588 (76.6%)	637/2991 (21.3%)	2354/2991 (78.7%)
Labeler 2	1357/6588 (20.6%)	5231/6588 (79.4%)	592/2991 (19.8%)	2399/2991 (80.2%)
Agreement between labelers	88.1%	96.4%	87.8%	95.4%

### Model training

The Training/Validation Dataset was used for model training. The dataset consisted of 201 patients with a total of 6588 individual axial images. The axial images and associated labels from the 201 patients were shuffled and divided into a training (4941 images) and validation cohort (1647 images).

Seven neural network configurations were trained until binary cross-entropy loss had not decreased for 10 epochs. Comparison of the trained models is shown in (Table 3). Model 6 achieved the highest classification accuracy and lowest binary cross-entropy loss on the validation dataset and was carried forward for further testing. Model 6 consisted of the ResNet-50 CNN with two fully connected layers with 512 neurons each and two dropout layers with 30% dropout (Fig. 3).

**Table 3:** Comparison of model performance during training.

Model Architecture	Epochs	Validation Accuracy	Validation Loss	Training Accuracy	Training Loss
Model 1	50	89.88%	0.4223	97.12%	0.0892
Model 2	50	90.95%	0.3093	99.13%	0.0219
Model 3	50	91.09%	0.3979	98.99%	0.0292
Model 4	54	91.53%	0.3562	99.10%	0.0260
Model 5	62	90.78%	0.2932	99.12%	0.0238
Model 6	77	92.41%	0.2569	99.03%	0.0284
Model 7	69	92.23%	0.2593	99.23%	0.0234

### Model testing

**Figure 4:**
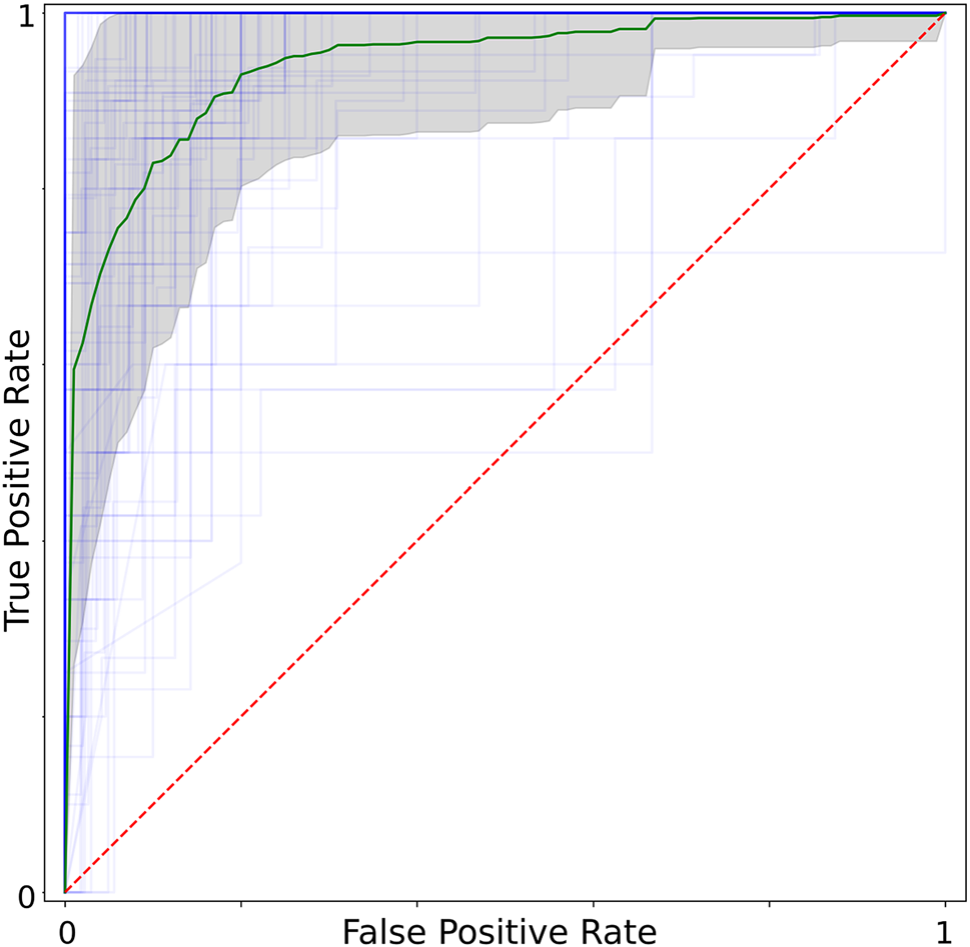
An area under the receiver operating characteristic curve plot showing model performance on each patient in the holdout dataset. The green curve represents the average ROC curve for all patients, while the light blue lines represent ROC curves for each individual patient. The gray region represents one standard deviation above and below the average curve.

The deep learning model was applied to the holdout dataset of 2991 images. On the entire holdout dataset the model achieved an AUC of 0.94, sensitivity of 0.88, specificity of 0.89, and f1-score of 0.82 (Fig. 4).

For each of the 88 patients in the holdout dataset the classification output of the deep learning model for each slice was compared to the class assigned by the human labelers. A ROC curve and AUC metric was generated for each patient by comparing the predicted and actual classes for each slice. The model achieved a median AUC of 0.94 on patients in the holdout dataset (Table 4). The model AUC in each subgroup was compared to the model performance on the entire holdout dataset (Table 4). The AUC in each subgroup did not significantly differ from the AUC in the entire holdout dataset, as evidenced by a p-value > 0.05 in each comparison.

**Table 4:** Model performance on the holdout dataset stratified by patient characteristics and MRI scanner manufacturer.

	Area Under the Curve (SD)	p-value
Entire Holdout Dataset (n = 88)	0.94 (0.08)	
**Age (years)**		
< 40 (n = 9)	0.88 (0.14)	0.12
40–65 (n = 63)	0.95 (0.06)	0.78
> 65 (n = 16)	0.92 (0.09)	0.45
**mJOA**		
18 (n = 2)	1.00 (0)	0.94
15–17 (n = 22)	0.96 (0.04)	0.67
12–14 (n = 39)	0.92 (0.09)	0.62
< 12 (n = 25)	0.95 (0.07)	0.77
**MRI Scanner Manufacturer**		
GE Medical Systems (n = 52)	0.94 (0.07)	0.82
Siemens (n = 25)	0.93 (0.06)	0.71
Philips Medical Systems (n = 11)	0.95 (0.08)	0.74
**Location**		
North America (n = 33)	0.95 (0.07)	0.68
South America (n = 16)	0.93 (0.09)	0.67
Europe (n = 21)	0.91 (0.08)	0.78
Asia Pacific (n = 18)	0.93 (0.07)	0.81

### Model interpretation and data visualization

**Figure 5:**
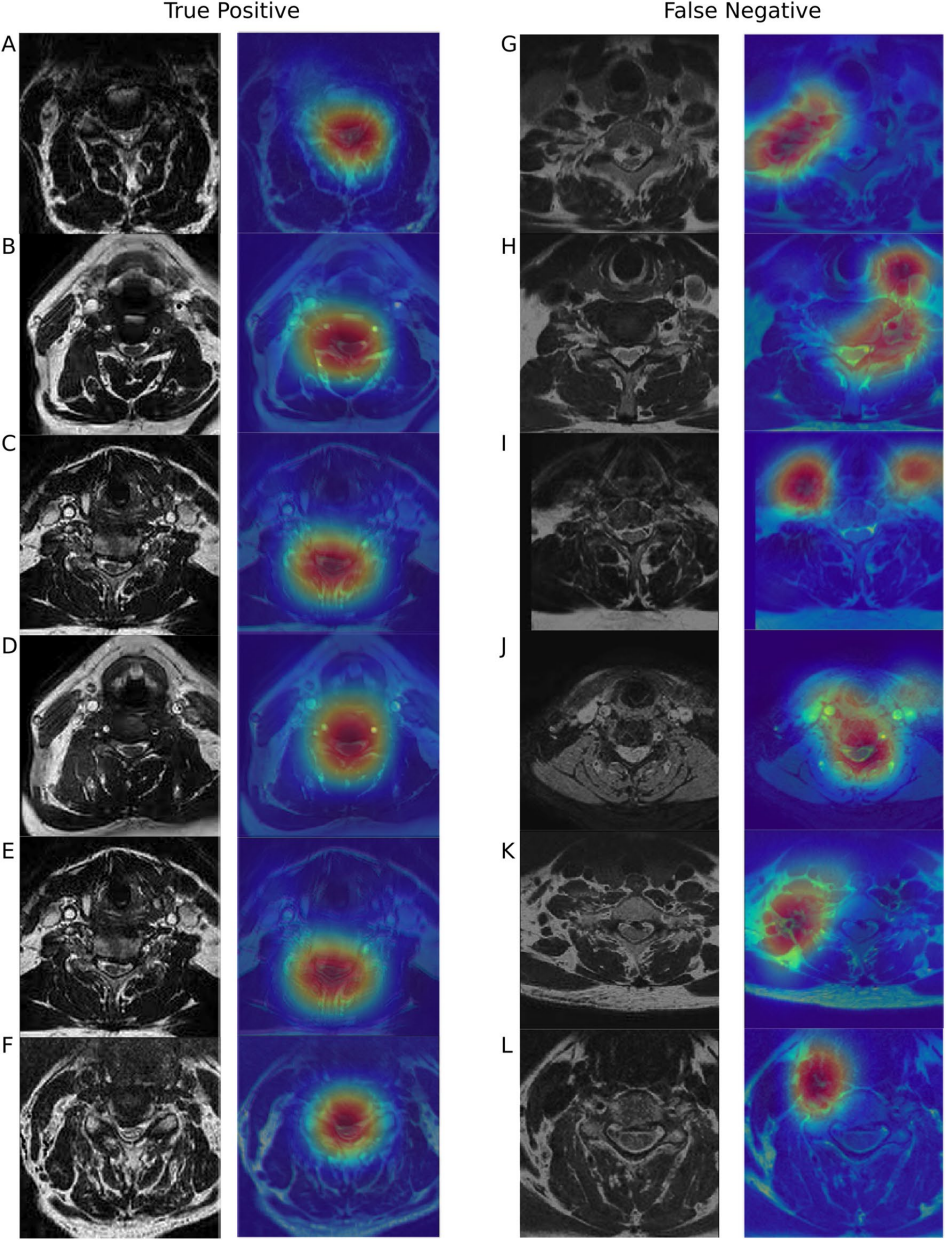
Class activation maps for example images that were classified correctly (true positives) or incorrectly (false negatives). The first column represents the axial T2-weighted MRI image. The second column shows the MRI image with the class activation map overlaid. In the class activation maps blue represents no activation while red represents maximal activation. On these example images of true positive classifications, the maximal activation tended to be over the spinal canal and spinal cord (Images **A**,**B**,**C**,**D**,**E**,**F**). In the false negative classifications, the activation was sometimes over irrelevant areas of the image, such as the paraspinal muscles or vascular structures (Images **G**,**I**,**K**,**L**). In other examples of false negative classifications there was activation over the spinal cord and spinal canal (Images **H** and **J**), but in these cases there was also activation over other seemingly irrelevant areas of the image.

Figure 5 depicts the class activation maps for randomly selected examples of true positive and false negative predictions by the model. These images were examined to gain insight into the features that corresponded to activation of the compressed or not-compressed class. In the example images of true positive classifications, the CAMs appeared to show activation over clinically relevant areas of the image such as the spinal cord and CSF spaces (Fig. 5). In the example images of false negative classifications, in some cases the model appeared to rely upon features outside of the spinal canal, such as the para-spinal muscles or vascular structures, for its predictions. In other cases, among the false negative images the model did appear to focus on clinically relevant areas of the image such as the spinal cord and CSF spaces, but this still resulted in an incorrect classification.

## Discussion

In this study, we trained and tested a CNN model for detection of spinal cord compression in cervical spine MRI scans using a dataset of 289 patients with DCM. We demonstrated the feasibility of training an existing CNN model for a novel medical imaging classification task. The model performed well on our holdout dataset (AUC = 0.95) and performed well across differing subgroups of patients and scanner types. To our knowledge, this study is the first to leverage a large prospective dataset of cervical spine MRI scans and to produce a model with high accuracy for detecting spinal cord compression. This model has potential utility in the context of a clinical trial to provide rapid automated coding of MRI scans of the cervical spine. In the future this model could be used to increase the efficiency and objectivity of interpreting cervical spine MRI scans.

There is increasing recognition that machine learning techniques that make use of CNNs will play an important role in medical diagnostics in the future^5,6,31,32^. While systems making use of CNNs have been developed and received regulatory approval in brain trauma, mammography, and chest radiographs, the use of these techniques to provide automated interpretation of imaging in spinal disorders is less developed^11,33–35^. The majority of studies that are comparable to ours have focused on classifying MRI images of the lumbar spine^13,36–38^. A study by Jamaludin et al. made use of the MRI images of 12,018 lumbar intervertebral discs from 2009 patients and developed a CNN to classify lumbar disc degeneration, disc narrowing, upper/lower endplate defects, upper/lower marrow changes, spondylolisthesis, and central canal stenosis^39^. These researchers used the VGG convolutional neural network and trained this model with randomly initialized weights instead of using transfer learning as we did in our study^40^. The DeepSPINE framework was developed using a large dataset of 22,796 MRI images of the lumbar spine from 4075 patients^41^. This study trained a CNN to classify lumbar canal stenosis and foraminal stenosis and achieved accuracy of 84.5% at grading lumbar canal stenosis. These authors made use of a segmentation algorithm that segmented the vertebral bodies as well as intervertebral disks and fed the output of this model into the ResNet-50 CNN for classifications. This differs from our approach, as we did not use a separate segmentation step and instead trained the ResNet-50 CNN using axial T2 weighted images of the entire cervical spine. Image segmentation can potentially allow for automated localization of pathologic findings, which was not possible with the simpler approach that we took. Another study by Lewandrowski et al. used a dataset of lumbar spine MRI scans from 3560 patients and developed a model to grade lumbar disc herniation and canal stenosis^42,43^. Similar to the DeepSpine framework, their approach consisted of a segmentation step in which intervertebral disks were segmented. This was followed by a custom CNN that graded the amount of disk herniation and canal stenosis. These authors achieved an AUC of 0.808 for detection of disk herniation. This approach differs from ours as we did not use a segmentation step and we did not grade the severity of spinal cord compression, but rather used a binary classification system of compressed vs. not-compressed.

Most previously published work on automated analysis of degenerative spine imaging has focused on the lumbar spine but some published reports have applied deep learning models to cervical spine images. Weber et al. trained a CNN to quantify the extent of fatty infiltration in MRI scans of the cervical spine and showed that model-generated parameters correlated with clinical measures such as neck pain and neck-related disability^15^. Jin et al. made use of an imaging dataset of diffusion tensor imaging (DTI) studies of patients that subsequently underwent surgery for DCM^14^. This group trained a ML model to predict positive surgical outcome with an accuracy of 88.62% in their training cohort. But they were unable to adequately validate their model given the size of their cohort, which consisted of 35 patients.

During our data labeling process two labelers examined each image and assigned a label of compressed or not-compressed. Agreement between the two labelers was good with a Cohen’s Kappa of 0.82 and 0.83 on the training/validation and holdout dataset, respectively. Assessing spinal cord compression can be difficult because although objective criteria have been proposed, evaluation in a clinical setting is typically subjective^3^. The images where the two raters disagreed were typically images in which there were mild degenerative changes but the spinal cord was not significantly compressed. In these cases a final consensus label was decided upon after discussion. Future work in this area might try to develop a model to grade the severity of cervical spinal cord compression into multiple classes instead of a binary class as we did. To accomplish this, cervical spinal MRIs should be labelled using a grading system such as the one proposed by Kang et al^44^. In this system 4 grades of spinal cord compression are subjectively evaluated and assessment of inter-rate reliability showed a Cohen’s Kappa of 0.6. We used a simpler binary grading system and achieved a higher Cohen’s Kappa of 0.82–0.83.

During model training we tested a variety of model architectures. All models used the ResNet50 CNN architecture with pre-trained weights and the fully connected layers were varied between the models. We found that all the tested models performed well with accuracy ranging from 89.88% to 92.39%. The best performing model had two fully connected layers with 512 neurons each and two dropout layers with 30% dropout each. This model performed well on the holdout dataset with an AUC of 0.94 and F1-score of 0.82. These performance metrics are similar to the best performing lumbar spine models that have been previously published^39,41–43^.

We generated CAMs using example images from the testing dataset that were classified correctly (true positives) and incorrectly (false negatives). Class activation maps have limitations and can suffer from a lack of repeatability or reproducibility^45,46^. In particular, CAMs have been shown to have limited utility in localizing pathologic features in medical imaging data, and localization or segmentation models are preferred in this circumstance^45^. Even when CAMs are being used for post-hoc analysis of a model to highlight features that the model deemed relevant for prediction, CAMs are still limited in utility. Adebeyo et al. showed that when labels were randomly permuted after training a CAM still sometimes highlighted areas of the image that seemed visually plausible^46^. We interpret the results of our CAMs in light of these limitations. We examined CAMs for a small number of example images and found that in the true positive images the CAM seemed to highlight the spinal cord and CSF space, which are clinically relevant. In the false negative images, which represented a small minority of classifications, the CAMs often showed activation over irrelevant areas of the image such as vascular structures or paraspinal musculature. This may provide some evidence that the model is focusing on areas of the image that are clinically relevant to spinal cord compression as opposed to spurious features that might coexist with spinal cord compression. However, to confirm this would require examination of all testing images instead of a subset. In addition, the CAMs should be further analyzed to determine, repeatability, reproducibility, and sensitivity to model weight randomization, which is beyond the scope of this study^45^.

Our study has a number of limitations. Firstly, our dataset only included patients who had a confirmed diagnosis of DCM and went on to get surgery. Patients with mild DCM or normal MRI scans were therefore underrepresented in our dataset. Including asymptomatic patients or patients with mild DCM symptoms during model training could have resulted in a greater variety of training images and perhaps a more generalizable model. Many patients in our dataset did not have MRI scans that were in an appropriate format to be included, which limited the final number of images that we were able to use for model training. We made use of two data-labelers and reconciled differences between the data-labelers with a consensus process. The inclusion of a third rater would have increased the validity of our ground-truth set of labels and would have permitted better assessment of model performance. During data labelling and model training we did not distinguish between partial and circumferential spinal cord compression. In clinical practice, however, circumferential spinal cord compression is a more severe finding and is more likely to result in clinical symptoms. This choice limits the clinical utility of our model. Future work in this area might attempt to develop a model that can grade cervical spinal cord compression into > 2 categories, which would be more clinically useful.

We developed a CNN that can detect spinal cord compression in cervical spine MRI scans with high performance. Our model could have utility in its current form in the context of a clinical trial to automatically code MRI scans. In this context, the model could be used to automatically extract features such as the number of slices showing spinal cord compression from MRI scans gathered as part of a clinical trial, which could generate data to be used for secondary analyses. We acknowledge, however, that challenges remain before this model could be used in a clinical setting. Our model performs a narrow classification task. A more general model would be able to detect and distinguish between multiple pathologies such as foraminal stenosis, disc herniation, ossification of the posterior longitudinal ligament, spondylolisthesis, and ligamentous hypertrophy with high accuracy. Such a model would likely require a dataset of thousands of patients to develop and test. Other researchers have noted that the creation of large imaging datasets is a central challenge in the development of clinically-useful machine learning models^31,47–49^. Although challenges remain to be overcome, our results suggest it is feasible to train an existing CNN for a novel medical imaging classification task within spine surgery and our approach could be applied to develop a more general model with a larger training dataset.

## Conclusion

In recent years automated diagnostic tools have reached a substantial level of development and this progress is expected to continue. In this study we trained and tested a CNN model to detect spinal cord compression in cervical spine MRI scans. We achieved high model performance with an AUC of 0.94 on a heterogenous group of patients. We demonstrated the feasibility of training an existing CNN for a novel medical imaging classification task. Future work will need to focus on developing larger datasets to facilitate the development of a more generalized model capable of quantifying cord signal change, severity of spinal cord compression, cervical spine deformity, and nerve root compression. A more generalized model may be able to improve radiology workflows and augment clinical decision-making by increasing the efficiency and objectivity of cervical spine MRI interpretation.

## Supplementary Information

Supplementary Information.
