# Supplementary material for: A deep learning model for detection of cervical spinal cord compression in MRI scans
Source: Sci Rep. 2021 May 18;11:10473. doi: 10.1038/s41598-021-89848-3 (PMC8131597; doi:10.1038/s41598-021-89848-3)
Supplement: Supplementary file 1 — Supplementary Information. [file 41598_2021_89848_MOESM1_ESM.pdf]

A Deep Learning Model for Detection of Cervical Spinal Cord Compression in MRI Scans

Supplementary Material

Zamir Merali, M.D [1], Justin Wang, M.D [1], Jetan H. Badhiwala, M.D [1], Christopher D. Witiw, M.D, MSc., FRCSC [1,2], Jefferson R. Wilson, M.D, PhD., FRCSC [1,2], Michael G. Fehlings, M.D, PhD., FRCSC [1,3]

1. Division of Neurosurgery, University of Toronto, Toronto, Ontario, Canada
2. Division of Neurosurgery, St. Michael's Hospital, Toronto, Ontario, Canada
3. Division of Neurosurgery, Toronto Western Hospital, Toronto, Ontario, Canada

Supplemental Methods

Table S1 – Overview of institutional review boards involved in NCT00285337, NCT00565734 clinical trials.

Location	Institution(s)
Thornton, Colorado, United States, 80229	Spine Education and Research Institute, Research Ethics Board
Atlanta, Georgia, Unites States, 30329	Emory University, Office of Research Compliance
Indianapolis, Indiana, United States, 46260	Indiana Spine Group, Institutional Review Board
Kansas City, Kansas, United States, 66160	Kansas University Medical Center, Research Institute
Baltimore, Maryland, United States, 21287	John Hopkins University, Institutional Review Board
Boston, Massachusetts, United States, 02115	Brigham and Women's Hospital, Partners Human Research Committee<br>New England Baptist Hospital, Institutional Review Board
Rochester, Minnesota, United States, 55905	Mayo Clinic, Institutional Review Board
Philadelphia, Pennsylvania, United States, 19107	Thomas Jefferson University and Rothman Institute Orthopaedics
Salt Lake City, Utah, United States, 84108	University of Utah, Institutional Review Board
Charlottesville, Virginia, United States, 22908	University of Virginia, Institutional Review Board
Seattle, Washington, United States, 98104	Harborview Medical Center, Institutional Review Board
Toronto, Ontario, Canada, M5T2S8	University of Toronto University Health Network, Research Ethics Board

Supplemental Figures

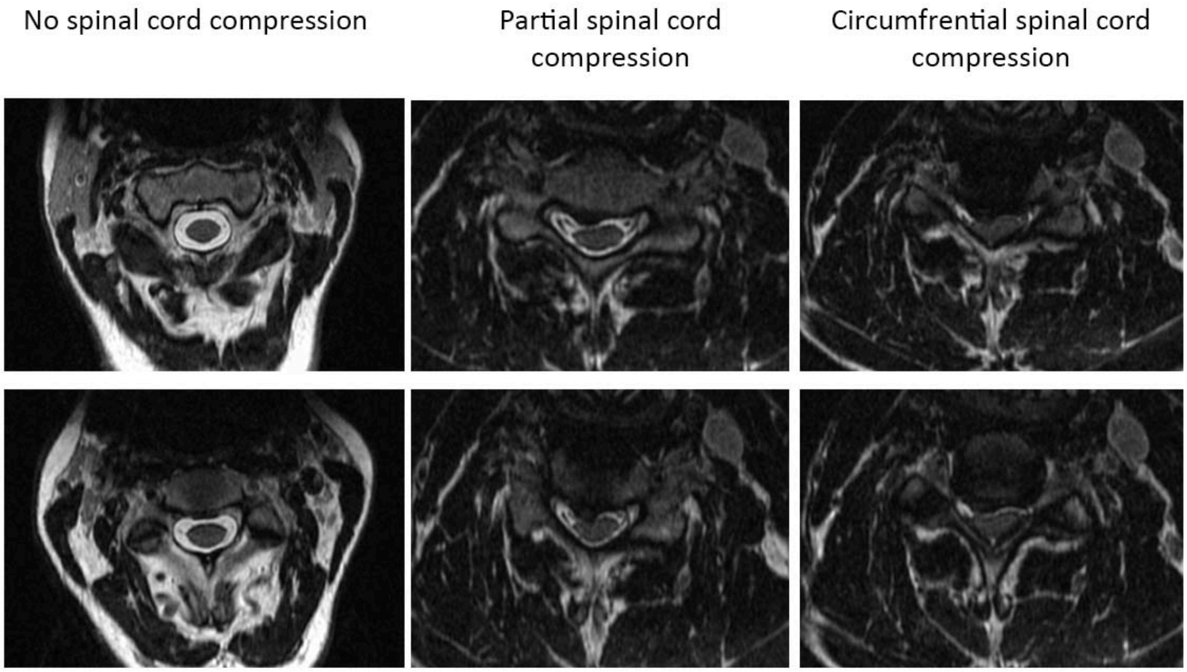

Figure S1 – Representative images showing no spinal cord compression, partial spinal cord compression, or circumferential spinal cord compression.
